# Primary Endpoint and Analysis Model for Prevention Trials with Participants with Preclinical AD: Lessons Learned from the DIAN‐TU Platform Trial

**DOI:** 10.1002/alz70859_099564

**Published:** 2025-12-25

**Authors:** Yan Li, Guoqiao Wang

**Affiliations:** ^1^ Washington University in St. Louis, St. Louis, MO USA; ^2^ Washington University School of Medicine, St Louis, MO USA

## Abstract

**Background:**

With the emerge of effective treatments for mild cognitive impairment and moderate Alzheimer's disease (AD), there's a shift towards evaluating these interventions in individuals with preclinical AD or those with elevated amyloid levels in prevention trials. These participants, typically asymptomatic with a CDR global score of 0 at baseline, are assessed using endpoints like a cognitive composite or time‐to‐first‐event based on an increase in CDR global score. However, the subtle cognitive decline in the preclinical phase poses significant challenges in detecting treatment effects, as seen in the DIAN‐TU trial. Time‐to‐first‐event might not fully reflect treatment benefits throughout the trial duration, particularly as amyloid levels might not be normalized quickly enough to prevent progression. The DIAN‐TU platform trial, being one of the initial prevention trials for AD, has led us to propose alternative endpoints and analysis models based on the insights gained from this study.

**Method:**

We suggest adopting a time‐to‐recurrent‐event as the primary endpoint in preclinical AD trials, where events are defined by changes in CDR global or CDR‐SB scores. We propose analyzing these using Cox proportional hazards or frailty survival models. Additionally, we explore the use of blood‐based biomarker (BBB) enrichment design to stratify participants into cohorts with varying disease progression rates, enhancing power through stratified survival analysis or multiple‐part mixed model repeated measures (MMRM).

**Result:**

Using time‐to‐recurrent‐event based on CDR metrics captures treatment effects more comprehensively over the trial's duration compared to time‐to‐first‐event, leading to increased statistical power. The combination of BBB enrichment with the stratified analysis strategy further amplifies this power compared to trials without such enrichment or analyzed without stratification. Simulation results and comparisons between analyses results using time‐to‐recurrent‐progression and time‐to first‐progression in CDR global and CDR‐SB based on DIAN‐TU data will be presented.

**Conclusion:**

Our experience from the DIAN‐TU platform trial suggests that time‐to‐recurrent‐event is a more effective endpoint for preclinical AD trials. Survival analysis provides more tangible and clinically relevant insights by translating treatment effects into reductions in hazard rates or extended survival times.

